# On the use of multiple imputation to address data missing by design as well as unintended missing data in case-cohort studies with a binary endpoint

**DOI:** 10.1186/s12874-023-02090-5

**Published:** 2023-12-07

**Authors:** Melissa Middleton, Cattram Nguyen, John B. Carlin, Margarita Moreno-Betancur, Katherine J. Lee

**Affiliations:** 1 Clinical Epidemiology & Biostatistics Unit, Murdoch Children’s Research Institute, Royal Children’s Hospital, Melbourne, Australia; 2 Department of Paediatrics, The University of Melbourne, 50 Flemington Rd, Parkville, VIC 3052 Australia

**Keywords:** Missing data, Multiple imputation, Case-cohort study, Simulation study, Inverse probability weighting

## Abstract

**Background:**

Case-cohort studies are conducted within cohort studies, with the defining feature that collection of exposure data is limited to a subset of the cohort, leading to a large proportion of missing data by design. Standard analysis uses inverse probability weighting (IPW) to address this intended missing data, but little research has been conducted into how best to perform analysis when there is also unintended missingness. Multiple imputation (MI) has become a default standard for handling unintended missingness and is typically used in combination with IPW to handle the intended missingness due to the case-control sampling. Alternatively, MI could be used to handle both the intended and unintended missingness. While the performance of an MI-only approach has been investigated in the context of a case-cohort study with a time-to-event outcome, it is unclear how this approach performs with a binary outcome.

**Methods:**

We conducted a simulation study to assess and compare the performance of approaches using only MI, only IPW, and a combination of MI and IPW, for handling intended and unintended missingness in the case-cohort setting. We also applied the approaches to a case study.

**Results:**

Our results show that the combined approach is approximately unbiased for estimation of the exposure effect when the sample size is large, and was the least biased with small sample sizes, while MI-only and IPW-only exhibited larger biases in both sample size settings.

**Conclusions:**

These findings suggest that a combined MI/IPW approach should be preferred to handle intended and unintended missing data in case-cohort studies with binary outcomes.

**Supplementary Information:**

The online version contains supplementary material available at 10.1186/s12874-023-02090-5.

## Introduction

The case-cohort study design provides a powerful and cost-effective variation on the standard cohort study when the exposure is costly to measure, for example when it involves metabolite levels [1]. In this design, a subcohort is randomly selected from the main cohort and the expensive exposure information is only collected on the participants within the subcohort and on cases of the primary outcome, noting that some subcohort members may also be cases. Hereinafter we refer to the subcohort and cases collectively as the study ‘subset’. Analysis is generally conducted on this subset, with the exposure intended to be missing ‘by design’ in the remainder of the cohort.

In such a design, it is important that the analysis accounts for the resulting unequal sampling probabilities due to all cases being selected into the subset (probability of selection = 1) and non-case subcohort members selected with a probability < 1 [2]. Standard practice is to use inverse probability weighting (IPW) to account for this unequal sampling [3]. IPW involves discarding observations with missing exposure data (i.e. those not in the subset) and weighting the remaining observations in the analysis by the inverse probability of selection, to not only represent themselves, but also those not selected into the subset [4].

As with any study, it is common to have missing data due to non-response in several study variables (e.g. exposure and/or covariates). We will refer to this as unintended missing data. A popular approach to handling unintended missing data is multiple imputation (MI). MI is a two-stage process. In the first stage, imputed values are drawn from an approximate posterior distribution for the missing values dependent on the observed data [5]. Values are imputed several times to form *m* completed datasets. In the second stage, each completed dataset is analysed using the target analysis model and results are pooled across the* m* datasets using Rubin’s rules to obtain an overall estimate for the parameter of interest with an estimated variance [6]. For MI to produce unbiased estimates with correct standard errors (SE), the imputation model needs to be compatible with the analysis model [7, 8]. Simply put, this means the imputation model should include all variables and features of the analysis model. In the context of case-cohort studies analysed using IPW, and weighted analyses more broadly, this means accounting for the weights used in the analysis model within the imputation model [9, 10]. Previous work by the authors studied different approaches to account for weights in MI in the context of a binary endpoint, and found that inclusion of the weights in the imputation model results in valid inferences when using MI in combination with IPW to address the intended and unintended missing data respectively [11]. One question that was not considered in M Middleton, C Nguyen, M Moreno-Betancur, JB Carlin and KJ Lee [11] was whether MI of IPW alone could be used to address both the intended and unintended missing data in case-cohort studies, rather than the standard practice of using MI in combination with IPW.

The use of MI to handle intended missing data in case-cohort studies has previously been investigated in the context of a time-to-event outcome, where it was found to perform well provided the outcome and all variables in the analysis model were included in the imputation model [12–14]. However, these studies did not consider the scenario in which there are also unintended missing data. RH Keogh, SR Seaman, JW Bartlett and AM Wood [15] extended this work, comparing three approaches for using MI in a case-cohort setting with unintended missing data. They compared: the ‘substudy’ approach, which uses the subset only to fit an imputation model for unintended missing data and uses IPW to handle intended missing data; the ‘intermediate’ approach, which uses the full cohort to fit an imputation model for the unintended missing data, but limits the analysis to those within the subset and uses IPW to handle intended missing data; and the ‘full’ approach, which uses the full cohort for imputation of both intended and unintended missing data and conducts an (unweighted) analysis. They showed all approaches to have large gains in efficiency compared to a complete-case analysis (CCA), which conducts an unweighted analysis in participants with complete data only, with the full approach showing the largest gain. They did, however, find the intermediate approach to be more robust to misspecification of the imputation model than the full approach, which can be a concern when imputing the large proportion of intended missing information in case-cohort studies. A limitation of the RH Keogh, SR Seaman, JW Bartlett and AM Wood [15] study was that they only considered the scenario where each variable could either have intended or unintended missing data, but not both, a scenario that is likely to arise in practice. It was also restricted to time-to-event analyses. Case-cohort studies are also used in the context of a binary outcome with fixed follow-up time [14, 16], which was not considered by RH Keogh, SR Seaman, JW Bartlett and AM Wood [15].

In the current study, we aimed to address these gaps by evaluating MI for handling both intended and unintended missing data in the exposure and/or confounders compared to the more standard MI/IPW approach, in the context of a case-cohort analysis of a binary outcome. We considered the substudy, intermediate and full MI approaches, introduced by RH Keogh, SR Seaman, JW Bartlett and AM Wood [15] as well as an IPW-only and CCA (5 approaches in total).

The paper is structured as follows. We first introduce a motivating example from the Barwon Infant Study (BIS), a birth cohort study in Victoria, Australia, and then describe the approaches for handling intended and unintended missingness in the case-cohort design that we compared. We then provide details of our simulation study, which was based on the motivating example and describe the application of the analysis approaches to the case study. We then present the results from the simulation and the case studies. We conclude with a discussion and recommendations for practice.

## Methods

### Case study

The motivating example for this manuscript comes from BIS, which is a population-derived birth cohort study of 1,074 infants born in the Barwon region of Victoria, Australia. The cohort profile and study design have been described elsewhere [17]. Due to the costly nature of biosample analysis, BIS has adopted the case-cohort design in several investigations of exposure effects on outcomes. The empirical investigation of interest here focusses on the association between vitamin D insufficiency (VDI) at birth, measured as 25(OH)D_3_ serum metabolite levels below 50nM from cord blood, and the risk of food allergy at one-year, as determined by a combination of a positive skin prick test and a positive food challenge to one of five common allergens (sesame, peanut, cow’s milk, egg and cashew) [18]. Of the infants who completed the one-year follow-up (*n*=894), all of the cases (*n*=61) and a random subcohort selected with a probability of 0.30 (*n*=324) were chosen for inclusion in the case-cohort study and had the exposure measured (noting some infants were in both). Of the 365 infants in the subset, VDI was only measured in 278 infants (76.2%), hence 23.8% of the subset had unintended missing data in the exposure.

The estimand of interest for the case study was the risk ratio (RR) for food allergy comparing those with VDI to those without. A standard outcome regression approach was used for its estimation, adjusting for family history of allergy (FamHx), “Caucasian” ethnicity (Eth), number of siblings (NSib), domestic pet ownership (PetOwn) and antenatal vitamin D supplement usage (AnteVD). Estimation used the modified Poisson regression approach with a logarithmic link and “robust” variance estimation (due to the known convergence issues with log-binomial regression [19, 20]) to fit the following model:

1 $$\mathrm{log}\left\{\mathrm{Pr}\left(\mathrm{FoodAllergy}=1\right)\right\}={\theta }_{0}+{\theta }_{1}\mathrm{VDI}+{\theta }_{2}\mathrm{Eth}+{\theta }_{3}\mathrm{FamHx}+ {\theta }_{4}\mathrm{PetOwn}+ {\theta }_{5}\mathrm{AnteVD}+ {\theta }_{6}I\left[\mathrm{NSib}=1\right]+{\theta }_{7}I\left[\mathrm{NSib}=2\right]$$

where $I\left[.\right]$ is an indicator function for the equality contained within the brackets (equal to 1 if the equality holds and 0 otherwise). The parameter of interest is $\mathrm{log}\left(\mathrm{RR}\right)={\theta }_{1}$. This is a slightly modified analysis to that used in the published version of this study, which used a log-binomial regression model to estimate the RR adjusted for a slightly different set of confounders. A description of the variables used for the current study can be found in Table 1, limited to participants with complete outcome data to align with the scope of this study.

**Table 1:** Description of variables in the Barwon Infant Study and their respective level of missingness in the full cohort and subset in participants with complete outcome data

**Variable**	**Label**	**Full cohort (*n*=786)**		**Subset (*n*=325)**	
		**Summary**	**Missing (%)**	**Summary**	**Missing (%)**
***Outcome***					
Food allergy at 1 year – *present – n(%)*	FoodAllergy	61 (7.8)	0 (0.0)	61 (18.8)	0 (0.0)
***Exposure***					
Vitamin D insufficiency at birth – *present – n(%)*	VDI	132 (45.1)	493 (62.7)	109 (44.3)	79 (24.3)
***Confounders***					
Ethnicity – *“Caucasian” – n(%)*	Eth	573 (73.2)	3 (0.4)	240 (74.3)	2 (0.6)
Domestic pet ownership – *present – n(%)*	PetOwn	602 (77.4)	8 (1.0)	239 (74.2)	3 (0.9)
Antenatal vitamin D usage – *present – n(%)*	AnteVD	460 (79.3)	206 (26.2)	193 (76.0)	71 (21.9)
History of family allergy – *present – n(%)*	FamHx	675 (86.9)	9 (1.2)	284 (88.2)	3 (0.9)
Number of siblings *– n(%)*	NSib		0 (0.0)		0 (0.0)
None		320 (40.7)		113 (34.8)	
One		281 (35.8)		130 (40.0)	
Two or more		185 (23.5)		82 (25.2)	
***Auxiliary***					
Maternal age at birth (*years*) – *mean(SD)*	MAge	32.1 (4.8)	3 (0.3)	33.0 (4.3)	0 (0.0)
SEIFA tertile *– n(%)*	SEIFA		14 (1.8)		6 (1.9)
Low		172 (22.3)		77 (24.1)	
Middle		148 (19.2)		61 (19.1)	
High		452 (58.6)		181 (56.7)	

*SEIFA* Socioeconomic Index for Area

### Analysis methods to account for the missing data

Below we outline the approaches we considered for the handling of missing data in the analysis of case-cohort studies that have unintended missing data in the exposure and confounders. We comment on alternative approaches that we could have considered in the discussion.

#### MI-based approaches

We considered the three MI-based approaches as proposed by RH Keogh, SR Seaman, JW Bartlett and AM Wood [15]:

i. Subset (*MI-IPW-Sub*) – Subset data are used to fit an imputation model addressing the unintended missing data. The imputed datasets are analysed using a weighted regression model (to address the intended missing data), with the weights equal to the inverse probability of being selected into the subset
ii. Intermediate (*MI-IPW-Int*) – The full cohort is used to fit an imputation model to address the unintended missing data. This approach involves also imputing the intended missing exposure data. However, the analysis is limited to observations within the subset only (i.e. non-subset imputed records are discarded) and a weighted analysis is performed on the subset, with the weights equal to the inverse probability of being selected into the subset
iii. Full (*MI-only*) – the full cohort is used to fit an imputation model imputing both the intended and unintended missing data, with an unweighted analysis performed on the full cohort (i.e. MI is used to handle both the intended and unintended missing data).

For all MI approaches, the imputation model included the outcome, exposure, confounders and two auxiliary variables (included to improve the efficiency of MI [21]). The auxiliary variables were maternal age at birth and socioeconomic index for area (SEIFA) [22] tertiles. All incomplete variables (exposure and two confounders) were binary and were imputed using a fully parametric approach based on logistic regression models within the fully conditional specification framework [23]. Fifty imputed datasets were generated in each case.

Under the subset and intermediate approaches, the analysis model used IPW, with weights equal to the inverse probability of selection into the subset. The sampling weights for the *i*th observation, ${w}_{i}$ , are defined as:

2 $${w}_{i}={\mathrm{Pr}\left({S}_{i}=1|{Y}_{i}\right)}^{-1}$$

where ${S}_{i}$ is an indicator for subset membership and ${Y}_{i}$ the outcome, for the *i*th individual.

Given all cases are included in the subset, the weights in expression (2) are 1 for cases. For non-case subcohort members, expression (2) is the inverse probability of subcohort membership for non-cases, estimated by $\widehat{{w}_{i}}={\left({m}_{0}/{n}_{0}\right)}^{-1}$ where ${n}_{0}$ is the number of non-cases in the full cohort and ${m}_{0}$ the number of non-cases in the subcohort [24].

Including the weights as a covariate in the imputation model has shown good performance in minimising bias from an incompatible imputation model in a general weighting setting [25]. In the case-cohort setting, given the weights are constant within strata defined by the outcome, inclusion of the outcome in the imputation model, as is standard practice when using MI, is equivalent to including the weights as a covariate in the imputation model. And indeed, this approach has shown good performance in the case-cohort setting [11]. Therefore, for both the subset and intermediate MI approaches, the weights were incorporated via inclusion of the outcome as a predictor in each of the univariate imputation models within the FCS procedure [11].

#### IPW to handle intended and unintended missingness (IPW-only)

For completeness, we also considered a fully weighted approach. Here an IPW analysis was conducted on the complete records only, with weights representing the inverse probability of being a complete record, that is, records selected for the subset with complete data for all analysis variables.

The probability of being a complete record can be decomposed into the unintended and intended missingness probability components, assuming independence between the response indicator, ${R}_{i}$, and subcohort selection, ${S}_{i}$, given the outcome and the observed predictors of missingness (a plausible assumption), and independence between the observed predictors of missingness, ${{\varvec{Z}}}_{i}$, and subcohort selection, ${S}_{i}$, given the outcome:

3 $$\mathrm{Pr}\left({R}_{i}=1 \& {S}_{i}=1 \right|{Y}_{i},{{\varvec{Z}}}_{{\varvec{i}}})=\mathrm{Pr}\left({R}_{i}=1|{Y}_{i},{{\varvec{Z}}}_{{\varvec{i}}}\right)\mathrm{Pr}\left({S}_{i}=1|{Y}_{i}\right)$$

where ${R}_{i}$ is equal to 1 if all analysis variables have complete data (i.e. no unintended exposure or confounder missing data) and 0 otherwise (i.e. have any variable with unintended missing data), and ${{\varvec{Z}}}_{i}$ a set of completely observed predictors of (unintended) missingness that can include, but is not limited to, the analysis variables.

For the *IPW-only* approach, the probability of not having unintended missing data, $\mathrm{Pr}\left({R}_{i}=1|{Y}_{i},{{\varvec{Z}}}_{{\varvec{i}}}\right)$, was estimated by fitting a logistic regression model conditional on fully observed predictors of (unintended) missingness to the available data.

A weight for the *i*th individual was then estimated by combining the sampling weight estimate and the inverse estimated probability of being a complete observation from the logistic model: $\widehat{{u}_{i}}=\widehat{{w}_{i}}\times {\left\{\mathrm{Pr}\widehat{\left({R}_{i}=1|{Y}_{i},{{\varvec{Z}}}_{{\varvec{i}}}\right)}\right\}}^{-1}$. 

#### Complete case analysis (CCA)

For comparison, a CCA was also conducted, where observations with unintended missingness were deleted and IPW applied to the subset, using the sampling weights $\widehat{{w}_{i}}$, to address the intended missing data.

A summary of the analysis approaches is displayed in Table 2.

**Table 2:** Summary of analysis approaches used in the simulation and case studies

**Approach**	**Imputation Approach**	**Weights in analysis model**	**How missing data addressed**		**Imputation sample**	**Analysis sample**
			**‘by design’**	**‘by chance’**		
CCA	None	sampling	IPW	Not addressed	N/A	Subset
IPW-Only	None	combined^a^	IPW	IPW	N/A	Subset
MI-IPW-Sub	FCS with outcome only	sampling	IPW	MI	Subset	Subset
MI-IPW-Int	FCS with outcome only	sampling	IPW	MI	Full cohort	Subset
MI-Only	FCS imputation	None	MI	MI	Full cohort	Full cohort

*FCS* Fully conditional specification, *IPW* Inverse probability weighting, *MI* Multiple imputation

^a^Combined weights are the product of inverse probability of selection into subcohort and inverse probability of being a complete observation

### Simulation study

A simulation study was conducted to compare the performance of the full MI approach, two combined MI/IPW approaches, full IPW approach and a CCA for analysing case-cohort studies with a binary outcome where there is unintended and intended missing data, across a range of realistic scenarios. A complete-data analysis was also conducted, where an unweighted regression model was fitted to the simulated data prior to subcohort selection and missing data being induced, as a check of the data-generation process.

#### Data generation mechanisms

Three scenarios were considered with respect to the full cohort sample size and the probability of subcohort selection. The first approximately replicates BIS, with a full cohort of 1,000 and a subcohort selection probability of 0.3. We also consider scenarios with a full cohort of 10,000, and a subcohort selection probability of either 0.1 or 0.2, mirroring the large sample sizes and smaller selection probabilities of other studies [12, 13].

**Fig. 1:**
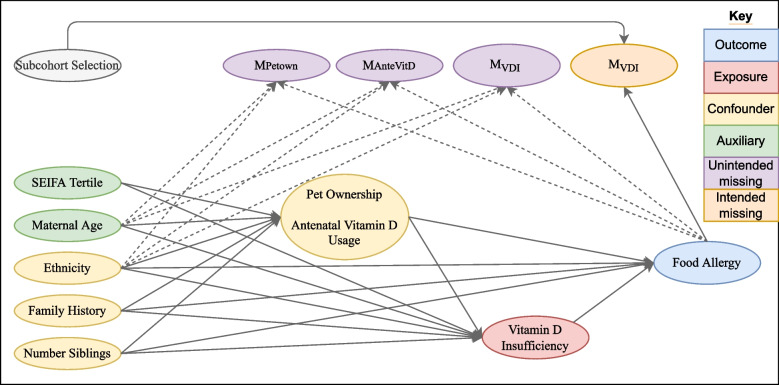
Missingness directed acyclic graph (m-DAG) depicting the assumed causal relationships between generated variables and their missingness indicators. Dashed lines represent associations present under the dependent missing data mechanism, but not the independent missing data mechanism

Complete cohorts were first generated based on plausible causal relationships between the relevant variables and their missingness indicators as shown in Fig. 1.

The exposure, five confounders and two auxiliary variables were generated in a sequential manner using the models below:

i. Ethnicity

4 $$\mathrm{Eth} \sim \mathrm{Bernoulli}\left(p\right)$$

ii. Maternal age at birth

5 $$\mathrm{MAge}={\delta }_{0}+{\delta }_{1} Eth+\epsilon$$

where $$\epsilon \sim N\left(0, {\sigma }^{2}\right)$$

iii. SEIFA tertile

6 $$\mathrm{log}\left\{\mathrm{Pr}\left(\frac{\mathrm{SEIFA}=1}{\mathrm{SEIFA}=0}\right)\right\}={\upzeta }_{0}+{\upzeta }_{1}\mathrm{ MAge}+{\zeta }_{2} \mathrm{Eth}$$

7 $$\mathrm{log}\left\{\mathrm{Pr}\left(\frac{\mathrm{SEIFA}=2}{\mathrm{SEIFA}=0}\right)\right\}={\eta }_{0}+{\eta }_{0}\mathrm{ MAge}+{\eta }_{0}\mathrm{ Eth}$$

iv. History of family allergy

8 $$\mathrm{logit}\left\{\mathrm{Pr}\left(\mathrm{FamHx}=1\right)\right\}={\iota }_{0}+{\iota }_{1} \mathrm{Eth}$$

v. Number of siblings

9 $$\mathrm{log}\left\{\mathrm{Pr}\left(\frac{\mathrm{NSib}=1}{\mathrm{NSib}=0}\right)\right\}={\kappa }_{0}+{\upkappa }_{1}\mathrm{MAge}+{\kappa }_{2} \mathrm{Eth}+ {\kappa }_{3 }I\left[\mathrm{SEIFA}=1\right]+{\kappa }_{4} I\left[\mathrm{SEIFA}=2\right]+ {\kappa }_{5 }\mathrm{FamHx}$$

10 $$\mathrm{log}\left\{\mathrm{Pr}\left(\frac{\mathrm{NSib}=2}{\mathrm{NSib}=0}\right)\right\}={\lambda }_{0}+{\lambda }_{1}\mathrm{ MAge}+{\lambda }_{2}\mathrm{ Eth}+ {\lambda }_{3} I\left[\mathrm{SEIFA}=1\right]+{\lambda }_{4} I\left[\mathrm{SEIFA}=2\right]+ {\lambda }_{5}\mathrm{ FamHx}$$

vi. Domestic Pet Ownership

11 $$\mathrm{logit}\{\mathrm{Pr}\left(\mathrm{PetOwn}=1\right)\}={\rho }_{0}+{\rho }_{1}\mathrm{ MAge}+{\rho }_{2} \mathrm{Eth}+ {\rho }_{3} I\left[\mathrm{SEIFA}=1\right]+{\rho }_{4} I\left[\mathrm{SEIFA}=2\right]+ {\rho }_{5} \mathrm{FamHx}+{\rho }_{5} I\left[\mathrm{NSib}=1\right]+ {\rho }_{6} I\left[\mathrm{NSib}=2\right]$$

vii. Antenatal vitamin D usage

12 $$\mathrm{logit}\{\mathrm{Pr}\left(\mathrm{AnteVD}=1\right)\}={\psi }_{0}+{\psi }_{1}\mathrm{ MAge}+{\psi }_{2} \mathrm{Eth}+{ \psi }_{3} I\left[\mathrm{SEIFA}=1\right]+{\psi }_{4} I\left[\mathrm{SEIFA}=2\right]+ {\psi }_{5}\mathrm{ FamHx}+{\psi }_{6} I\left[\mathrm{NSib}=1\right]+ {\psi }_{7} I\left[\mathrm{NSib}=2\right]$$

viii. Vitamin D insufficiency at birth,

13 $$\mathrm{logit}\left\{\mathrm{Pr}\left(\mathrm{VDI}=1\right)\right\}={\phi }_{0}+{\phi }_{1}\mathrm{ MAge}+{\phi }_{2} \mathrm{Eth}+ {\phi }_{3} I\left[\mathrm{SEIFA}=1\right]+{\phi }_{4} I\left[\mathrm{SEIFA}=2\right]+ {\phi }_{5}\mathrm{ FamHx}+{\phi }_{6} I\left[\mathrm{NSib}=1\right]+ {\phi }_{7} I\left[\mathrm{NSib}=2\right]+{\phi }_{8}\mathrm{ PetOwn}+ {\phi }_{9}\mathrm{ AnteVD}$$

Finally, the outcome was generated per the target analysis model (1). We varied the strength of the exposure-outcome association, and the associations between the auxiliary variable and the incomplete variables. Under ‘observed’ conditions, the associations were as estimated from the BIS case study, while under ‘enhanced’ conditions the exposure-outcome association was inflated to a RR of 2 (compared to RR=1.16 in BIS) and the associations of the exposure and missing confounders with the auxiliary variable maternal age were strengthened to represent an approximate 10-fold change in risk across the 30-year age range. An additional setting was considered, where the outcome generation model included an interaction between the exposure (VDI) and a confounder, ethnicity. This setting was designed such that the target analysis model was misspecified, as it excluded the interaction term, and enabled us to explore how the imputation models performed under a more complex but realistic scenario. The parameter values used for data generation under the various scenarios are given in the Supplementary Table S1.

Once the full cohort had been generated, unintended missing data were introduced into the two confounders, antenatal vitamin D usage and pet ownership, and the exposure. Two levels of missing data frequency were considered: low (20% of records in the full cohort had at least one confounder missing and 10% had unintended missing data in the exposure, with 25% of records having incomplete data), and high (percentages doubled).

Data were set to missing either using an independent missingness mechanism, where observations were randomly assigned to be missing with the desired proportions, or dependent on the outcome (expected to cause bias in the CCA), an auxiliary variable (expected to increase the efficiency of MI compared to CCA) and a confounder, as per Fig. 1. The degree of dependency between the missingness indicators was varied to control the overall proportion of missing data, and the distribution of missing data patterns. Under the dependent missingness mechanism, data were set to missing based on the following models (with parameter values given in the Supplementary Table S1):

14 $$\mathrm{logit} \left\{\mathrm{Pr}\left({\mathrm{M}}_{\mathrm{petown}}=1\right)\right\}={\nu }_{0}+{\nu }_{1}\mathrm{ FoodAllergy}+{\nu }_{2}\mathrm{ Eth}+{\nu }_{3 }\mathrm{Mage}$$

15 $$\mathrm{logit}\left\{\mathrm{Pr}\left({\mathrm{M}}_{\mathrm{ante}}=1\right)\right\}={\tau }_{0}+{\tau }_{1} \mathrm{FoodAllergy} +{\tau }_{2} \mathrm{Eth}+{\tau }_{3} \mathrm{MAge}+{\tau }_{4} {\mathrm{M}}_{\mathrm{petown}}$$

16 $$\mathrm{logit}\left\{\mathrm{Pr}\left({\mathrm{M}}_{\mathrm{vdi}}=1\right)\right\}={\omega }_{0}+{\omega }_{1}\mathrm{ FoodAllergy} +{\omega }_{2} \mathrm{Eth}+ {\omega }_{3}\mathrm{ MAge}+{\omega }_{4} I[{\mathrm{M}}_{\mathrm{petown}}=1 \left| {\mathrm{M}}_{\mathrm{antevd}}=1\right]$$

where ${\mathrm{M}}_{\mathrm{var}}$ is an indicator for missingness in variable “var”.

The strength of associations in the dependent missingness mechanism were varied, with the ‘observed’ scenarios using estimates from BIS as values for the regression coefficients of substantive predictors in models (14, 15 and 16), and the ‘enhanced’ scenarios doubling these coefficients. The values for ${\nu }_{0}$, ${\tau }_{0}$, ${\tau }_{4}$, ${\omega }_{0}$, and ${\omega }_{4}$ were iteratively chosen such that the desired proportions of missingness were achieved (see Supplementary Table S2).

Finally, the subcohort was randomly selected with the required probability of selection, and the exposure set to be missing in the non-subset members.

Altogether 26 scenarios were considered, comprised of 24 scenarios in a factorial design and an additional 2 scenarios where the interaction term was included in the data generation model. Scenarios are summarised in the Supplementary Table S3, and summary statistics for the simulated datasets provided in Supplementary Table S4.

#### Evaluation of analysis approaches

Each simulated dataset was analysed using each of the approaches for handling missing data to produce an estimate of the target parameter, the regression coefficient of the exposure in equation (1): $\mathrm{log}\left(\mathrm{RR}\right)={\theta }_{1}$.

Performance was evaluated using the relative bias (percentage bias relative to the true value of the target parameter, ${\theta }_{1}$), empirical and model-based SEs, and the coverage of the 95% confidence interval (CI) for the target parameter. In calculating these measures in the scenario where the analysis model was correctly specified (outcome generated from model (1)), the true value of the parameter of interest was the coefficient for the exposure used during outcome generation (${\theta }_{1}$). In scenarios where the analysis model was misspecified (outcome generated from a model including an exposure-confounder interaction), the true value was estimated as the average of the exposure coefficient estimates obtained when applying the target analysis model (model (1)) to 1,000 simulated populations of size 1,000,000. Monte Carlo standard errors (MCSE) are also reported.

A total of 2000 simulations were generated for each scenario, ensuring that the MCSE for a true coverage probability of 95% would be 0.49% [26]. All analyses were conducted in Stata 15.1 [27].

### Implementation of analysis methods in the case study

Each of the analysis approaches was applied to obtain estimates of the target parameter, ${\theta }_{1}$, in equation (1) in the case study. To align with the simulation study, the analysis was limited to observations with complete outcome data (full cohort *n* = 786, subset *n*=325). The incomplete variables in the case study were: VDI (414/786 intended missingness and 79/786 unintended missingness), pet ownership (1% missing in full cohort, 0.9% missing in subset), antenatal vitamin D usage (26.2% missing in full cohort, 21.9% missing in subset), history of family allergy (1.2% missing in full cohort, 0.9% missing in subset), Ethnicity (0.4% in full, 0.6% in subset), SEIFA tertiles (1.8% in full, 1.9% in subset), and maternal age (<0.01% in full). Binary variables were imputed using a logistic regression model, categorical variables using an ordinal logistic regression model, and continuous variables using a linear regression model. When the analysis approach required the use of sampling weights, the weight for the non-cases in the subcohort, were estimated using the proportion of non-cases selected for exposure measurement, i.e. ${\left(0.30\right)}^{-1}$.

## Results

### Simulation study

**Fig. 2:**
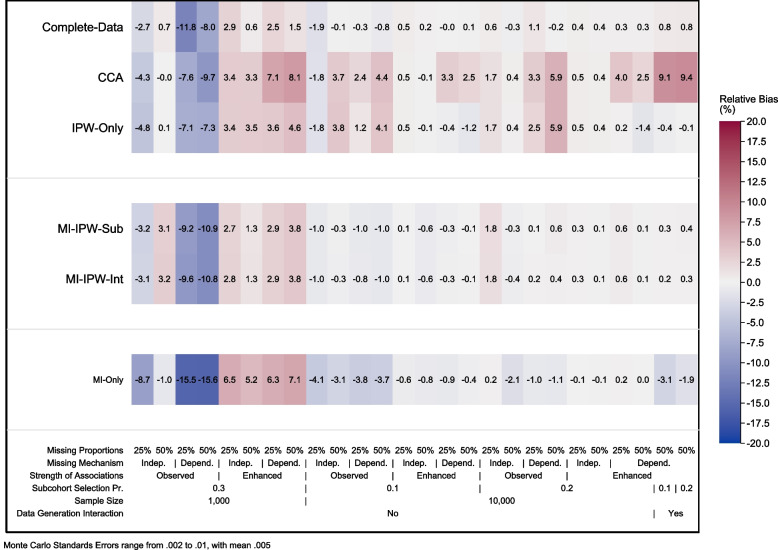
Relative bias (%) in the estimated coefficient for the target parameter across the 26 simulated scenarios

Figure 2 displays the relative bias for all approaches and scenarios. In all scenarios with a small sample size and an independent missingness mechanism, most approaches were approximately unbiased (<5%), with the exception of *MI-only* which showed some bias in scenarios with low levels of missing data (-8.7 – 6.5%). In all scenarios with a small sample size, dependent missingness mechanism, and an observed association, all approaches (including the complete data analysis) showed bias in the point estimate, with the largest biases for the *MI-only* approach (-15.6 – -15.5%). In contrast, when there was a small sample size with enhanced associations and dependent missingness, the *IPW-only*, subset and intermediate approaches were relatively unbiased (<4.6%), with slightly larger biases with the CCA and *MI-only* approaches (6.3 – 8.1%).

In all scenarios with a large sample size and correct specification of the analysis model, all approaches were approximately unbiased (<5.9%), with the largest biases for the CCA for the dependent missingness scenarios as expected. When the analysis model was misspecified (i.e. omitted the interaction term of the data generating model), the CCA was biased (9.1 – 9.4%) with all other approaches approximately unbiased (-3.1 – 0.4%).

**Fig. 3:**
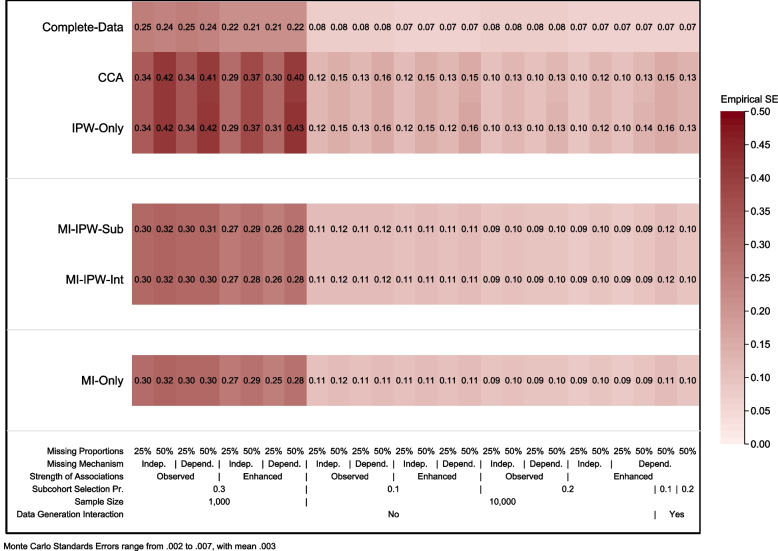
Empirical standard error for the target parameter for each of the 26 simulated scenarios

The empirical SE for all approaches and scenarios is presented in Fig. 3. *IPW-only* and CCA performed similarly in terms of their precision across all scenarios, with *IPW-only* tending to have a slightly lower precision in settings with a dependent missingness mechanism and a high proportion of missing data. The combined MI/IPW and *MI-only* approaches consistently showed a gain in precision (similar in magnitude for all approaches) compared to *IPW-only* and CCA. This gain in precision was greatest in scenarios with a sample size of 1,000 and a higher proportion of missing data. The relative error in estimating the SE for all methods and scenarios is presented in Supplementary Fig. S1 (see Supplementary Fig. S2 for the estimated model-based SE).

**Fig. 4:**
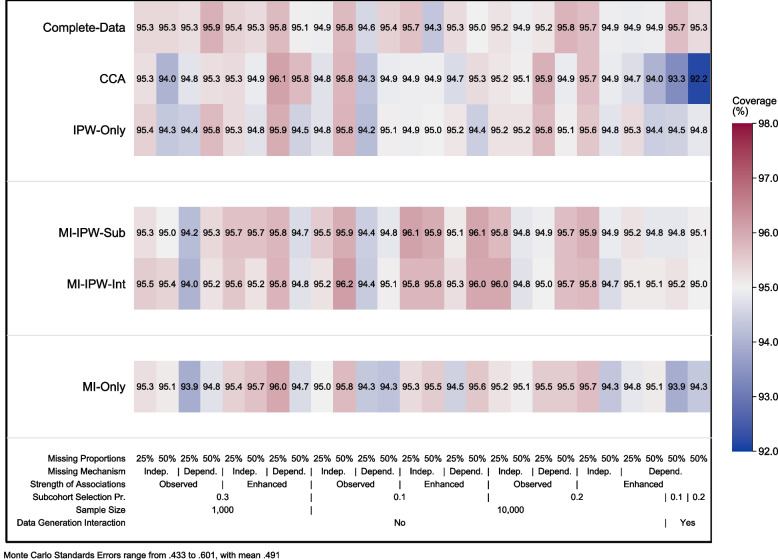
Coverage probability of the 95% confidence interval for each of the 26 simulated scenarios

The coverage probability of the 95% CI is shown in Fig. 4. Across all scenarios with correct specification of the analysis model the nominal coverage level of 95% was generally within the expected MCSE range for all approaches (93.9 – 96.2%), with the coverage probability closer to the expected probability of 95% as the sample size increased. When the analysis model was misspecified, the *MI-only* approach and CCA showed under-coverage, ranging from 92.2% to 94.3%, while the subset and intermediate MI approaches and the *IPW-only* approach showed close to the nominal coverage.

### Case study

**Fig. 5:**
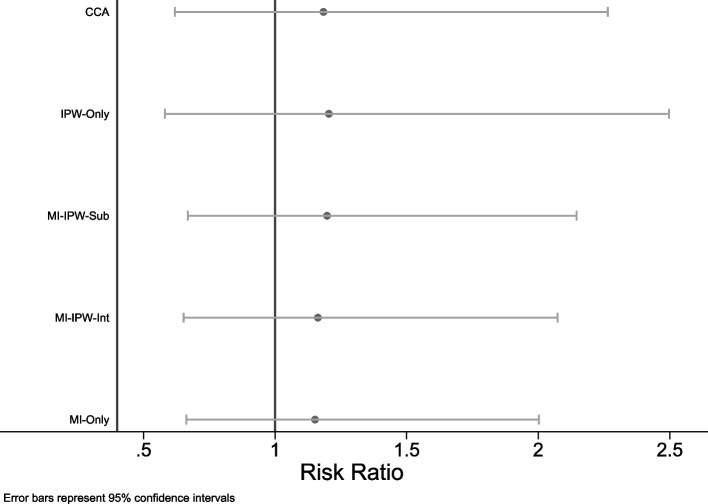
Estimated risk ratio and 95% confidence interval for the adjusted association between food allergy and vitamin D insufficiency estimated using the case study data

The estimated RR and its 95% CI obtained from applying each analysis method to the case study data are displayed in Fig. 5. All methods produced similar point estimates, suggesting an increasing risk of food allergy at 1-year for having VDI compared to not having VDI, however, there was a large amount of uncertainty in the true effect. The *MI-only* approach had a narrower CI compared to the MI/IPW approaches, with all approaches using MI having a narrower CI compared to the *CCA* and *IPW-only* approaches*.*

## Discussion

This study aimed to evaluate approaches to handling intended and unintended missing data in case-cohort studies with a binary endpoint. We conducted a simulation study to compare the performance of 5 analytic approaches (two MI/IPW approaches, a full imputation approach, a fully weighted approach and a CCA) across a range of scenarios.

When there was a small sample size, all analysis approaches, including the complete-data analysis, showed bias in the point estimate, which was not seen in scenarios with a large sample size. This is indicative of a finite sample bias in case-cohort studies, as previously observed by M Middleton, C Nguyen, M Moreno-Betancur, JB Carlin and KJ Lee [11] and RH Keogh, SR Seaman, JW Bartlett and AM Wood [15]. While the MI/IPW subset and intermediate approaches generally performed similarly to the complete-data analysis in these small-sample scenarios, larger biases were seen with the *MI-only* approach.

In settings where there was a large sample size, the combined MI/IPW approaches showed underestimation of the SE (and narrower CIs) in some settings. However, this did not translate into under-coverage of the 95% CI, and therefore may not warrant concern in practice. In the analysis model misspecification settings, the *IPW-only*, *MI-IPW-Sub* and *MI-IPW-Int* approaches showed consistently lower biases for both the point estimate and SE compared to *MI-only* and CCA. There was also no apparent gain in precision for using a full-MI approach compared to a combined MI/IPW approach under any scenario. Overall, these results suggest that combined MI/IPW may be the preferred approach, with little difference between the subset and intermediate approaches.

Previous work had suggested *MI-IPW-Sub* performed well in handling confounders with unintended missing values in case-cohort studies with binary outcomes [11]. The results presented in the current simulation study suggest that the good performance of this approach extends to scenarios where the exposure is missing “by chance” rather than by design. While MI provided some expected gains in the precision of the exposure-outcome effect compared to the *IPW-only* approach and CCA, the simulation study results showed no apparent gain in bias or precision using a full or intermediate MI approach over the subset MI approach. These results are in contrast to those presented by RH Keogh, SR Seaman, JW Bartlett and AM Wood [15] who found an intermediate MI approach provided greater gains in efficiency than a subset or full approach. It is important to note, however, that the subset approach may be subject to convergence issues in small case-cohort sample sizes, and an intermediate approach may be preferable in this setting. Interestingly, the *MI-only* approach tended to show slightly larger biases compared to the subset and intermediate MI approaches, suggesting a combined approach may be preferable.

It is important to note that in this paper we have only considered a single implementation of MI. In fact, MI is not a single approach, and decisions made during the set-up may impact the performance of the approach [21]. This impacts on the generalisability of our results, as a different implementation of MI may lead to different conclusions. However, our model was chosen to closely follow the data generation model and analysis model, and in this case we would expect MI to perform well.

A limitation of this paper is that we only considered incorporating the weights into the imputation model via inclusion of the outcome as this approach has shown to perform well in this setting [11]. Other approaches are available such as including the weights as a predictor in the imputation model along with all pairwise interactions between the weights and the covariates [9] and using a weighted imputation model. Another approach available to achieve imputation model compatibility is substantive model compatible fully conditional specification (smcfcs) [7]. However, at present, the smcfcs program in Stata and R cannot accommodate a weighted analysis model and hence was not considered in this study.

Our study was based on a realistic case-cohort setting and considered a large range of scenarios. While we considered a small number of scenarios where the analysis model was misspecified, further exploration is needed to assess the appropriateness of MI in such settings. Due to limitations in the handling of missing outcome data in case-cohort studies using weighting approaches, given the weights are derived dependent on the outcome status, we have not considered missing outcome data in this study. This provides an avenue for future work. Another limitation is that we only considered IPW, MI and combined MI/IPW approaches. There are alternative analysis approaches, such as the semiparametric maximum likelihood and improved weighting approaches, as presented by H Noma and S Tanaka [14], which could also be explored.

## Conclusions

Based on the findings in the current study, we conclude the combined MI/IPW approach may be preferable to a full MI approach to address both intended and unintended missing data in case-cohort studies with a binary endpoint, although the latter typically resulted in minimal bias and nominal coverage. The subset and intermediate combined approaches performed similarly, including in the scenarios where the analysis model was misspecified. Therefore, we recommend addressing unintended missing data through MI applied to either the subset or full cohort and addressing intended missing data through IPW (*MI-IPW-Sub, MI-IPW-Int)*.

### Supplementary Information

**Additional file 1:** Details pertaining to the data generation procedure and additional simulation results. **Supplementary Table S1.** Parameter values used in the generation of complete data and missing indicators, for observed and enhanced association scenarios. **Supplementary Table S2.** Iteratively chosen parameter values used to generate missing indicators for each data generation mechanism. **Supplementary Table S3.** Summary of the 26 scenarios considered in the simulation study. **Supplementary Table S4.** Summary statistics for the 26 scenarios, calculated across the 2,000 simulated datasets. Additional simulation study results. **Figure S1.** Relative error (%) in estimation of the standard error for the target parameter (comparison of empirical and model-based standard error) for each of the 26 simulated scenarios. **Figure S2.** Model-based standard error for the analysis approaches across the 2,000 simulated datasets under each scenario.

## Data Availability

The datasets used and/or analysed during the current study are available from the corresponding author on reasonable request.

## Abbreviations

BIS: Barwon Infant Study
CCA: complete-case analysis
CI: confidence interval
IPW: Inverse probability weighting
MCSE: Monte Carlo standard error
MI: Multiple imputation
RR: Risk ratio
SE: Standard error
SEIFA: Socio-economic index for areas
SMCFCS: Substantive model compatible fully conditional specification
VDI: Vitamin D insufficiency
