# Supplementary material for: On the use of multiple imputation to address data missing by design as well as unintended missing data in case-cohort studies with a binary endpoint
Source: BMC Med Res Methodol. 2023 Dec 7;23:287. doi: 10.1186/s12874-023-02090-5 (PMC10702035; doi:10.1186/s12874-023-02090-5)
Supplement: Supplementary file 1 — Additional file 1: Details pertaining to the data generation procedure and additional simulation results. Supplementary Table S1. Parameter values used in the generation of complete data and missing indicators, for observed and enhanced association scenarios. Supplementary Table S2. Iteratively chosen parameter values used to generate missing indicators for each data generation mechanism. Supplementary Table S3. Summary of the 26 scenarios considered in the simulation study. Supplementary Table S4. Summary statistics for the 26 scenarios, calculated across the 2,000 simulated datasets. Additional simulation study results. Figure S1. Relative error (%) in estimation of the standard error for the target parameter (comparison of empirical and model-based standard error) for each of the 26 simulated scenarios. Figure S2. Model-based standard error for the analysis approaches across the 2,000 simulated datasets under each scenario. [file 12874_2023_2090_MOESM1_ESM.docx]

**Supplementary material for manuscript titled: *Multiple imputation analysis of case-cohort studies with a binary endpoint***

Contents

[Data generation procedure 2](#_Toc145419753)

[**Supplementary Table S1:** Parameter values used in the generation of complete data and missing indicators, for observed and enhanced association scenarios. 3](#_Toc145419754)

[**Supplementary Table S2:** Iteratively chosen parameter values used to generate missing indicators for each data generation mechanism 4](#_Toc145419755)

[**Supplementary Table S3:** Summary of the 26 scenarios considered in the simulation study. 5](#_Toc145419756)

[**Supplementary Table S4:** Summary statistics for the 26 scenarios, calculated across the 2,000 simulated datasets. 6](#_Toc145419757)

[Additional simulation study results 7](#_Toc145419758)

[**Figure S1:** Relative error (%) in estimation of the standard error for the target parameter (comparison of empirical and model-based standard error) for each of the 26 simulated scenarios 8](#_Toc145419759)

[**Figure S2:** Model-based standard error for the analysis approaches across the 2,000 simulated datasets under each scenario 9](#_Toc145419760)

# Data generation procedure

Supplementary material provided in this section pertain to the data generation procedure of the simulation study as described in the manuscript.

Supplementary table S1 provides the exponentiated parameter values used to generate the complete data and the missing data indicators (1 if observation was incomplete for variable of interest and 0 is observation was complete). The table describes the model used for generation of each variable or indicator and provides the parameter value under each scenario. Parameter values under scenarios with observed association were chosen based on applying the data generation model to the case study data.

Supplementary table S2 provides the exponentiated parameter values for the parameters controlling the overall proportions of missing observations when modelling the missing indicator variables. Parameter values were chosen through an iterative process.

Supplementary table S3 provides a summary of the 26 scenarios considered in the simulation studies.

Supplementary table S4 provides summary measures for the 2,000 simulated datasets used for calculation of performance measures across the 26 scenarios. The outcome prevalence, case-cohort sample size (analysis sample used for subset and intermediate MI approaches), and of missing information are provided. The percentage of missing observations in the full cohort includes both the intentional and unintentional missing data and uses the sample size as the denominator. The percentage missing within the subset reflects the percentage of observations within unintentional missing data and uses the case-cohort sample size as the denominator. The first three letters of the scenario label reflect the strength of association, while the remaining letters reflect the missingness mechanism. Scenarios 1-3 have 25% incomplete observations, and scenarios 4-6 have 50% incomplete observations. Scenarios 1 and 4 have a cohort size of 1,000 and subcohort selection probability of 0.3, scenarios 2 and 5 have a cohort size of 10,000 and subcohort selection probability of 0.1, and scenarios 3 and 6 have a cohort size of 10,000 and a subcohort selection probability of 0.2. Scenarios ending in ‘x’ have an interaction term in the data generation model for the outcome.

**Supplementary Table S1:** Parameter values used in the generation of complete data and missing indicators, for observed and enhanced association scenarios.

| **Dependent Variable** | | **Model** | **Scenario** | **Intercept^** | *cauc* | *MAge* | *seifa=1* | *seifa=2* | *FamHx* | *NSib=1* | *NSib=2* | *PetOwn* | *AnteVD* | *VDI* | *VDI* $\times$ *cauc* | *FoodAllergy* |
| --- | --- | --- | --- | --- | --- | --- | --- | --- | --- | --- | --- | --- | --- | --- | --- | --- |
| *Cauc* | | Bernoulli | Both | 0.72 |  |  |  |  |  |  |  |  |  |  |  |  |
| *MAge* | | Linear# | Both | 31.33 | 1.01 |  |  |  |  |  |  |  |  |  |  |  |
| *SEIFA* | |  |  |  |  |  |  |  |  |  |  |  |  |  |  |  |
|  | *seifa=1* | Multinomial | Both | 0.10 | 1.65 | 1.05 |  |  |  |  |  |  |  |  |  |  |
|  | *seifa=2* |  | Both | 0.17 | 1.17 | 1.08 |  |  |  |  |  |  |  |  |  |  |
| *FamHx* | | Logistic | Both | 4.94 | 1.38 |  |  |  |  |  |  |  |  |  |  |  |
| *NSib* | |  |  |  |  |  |  |  |  |  |  |  |  |  |  |  |
|  | *NSib=1* | Multinomial | Both | 0.02 | 0.81 | 1.11 | 0.74 | 0.92 | 2.29 |  |  |  |  |  |  |  |
|  | *NSib=2* |  | Both | 0.0004 | 0.77 | 1.21 | 1.43 | 1.11 | 3.39 |  |  |  |  |  |  |  |
| *PetOwn* | | Logistic | Obs Assoc. | 11.84 | 1.17 | 0.97 | 1.33 | 0.74 | 0.83 | 1.11 | 1.36 |  |  |  |  |  |
|  |  |  | Enh Assoc. |  |  | 0.90 |  |  |  |  |  |  |  |  |  |  |
| *AnteVD* | | Logistic | Both | 0.03 | 1.36 | 1.16 | 1.11 | 0.98 | 1.49 | 0.51 | 0.28 |  |  |  |  |  |
| *VDI* | | Logistic | Obs Assoc. | 0.86 | 0.88 | 1.04 | 1.40 | 1.01 | 0.34 | 0.71 | 1.01 | 0.98 | 0.51 |  |  |  |
|  |  |  | Enh Assoc. |  |  | 1.11 |  |  |  |  |  |  |  |  |  |  |
| *FoodAllergy* | | Poisson | Obs Assoc. | 0.07 | 1.08 |  |  |  | 1.93 | 1.42 | 1.38 | 0.31 | 0.80 | 1.16 |  |  |
|  |  |  | Enh Assoc. |  |  |  |  |  |  |  |  |  |  | 2.00 |  |  |
|  |  |  | Interaction |  |  |  |  |  |  |  |  |  |  | 1.30 | 1.70 |  |
| *M_petown** | | Logistic | Obs Assoc. | 0.57 | 0.66 | 0.89 |  |  |  |  |  |  |  |  |  | 1.83 |
|  |  |  | Enh Assoc. |  |  | 0.90 |  |  |  |  |  |  |  |  |  |  |
| *M_antevd** | | Logistic | Obs Assoc. | 1.84 | 0.71 | 0.96 |  |  |  |  |  |  |  |  |  | 0.61 |
|  |  |  | Enh Assoc. |  |  | 0.90 |  |  |  |  |  |  |  |  |  |  |
| *M_vdi** | | Logistic | Obs Assoc. | 0.77 | 0.93 | 0.98 |  |  |  |  |  |  |  |  |  | 0.31 |
|  |  |  | Enh Assoc. |  |  | 0.90 |  |  |  |  |  |  |  |  |  |  |

*^Probability given for Bernoulli models, intercept for linear models, base odds for Logistic models and base risk for Poisson models
#Error terms were generated with a mean of 0 and standard deviation of 4.75
*Missing indicator models were additionally dependent on iteratively chosen values as shown in Table S2*

**Supplementary Table S2:** Iteratively chosen parameter values used to generate missing indicators for each data generation mechanism

| **Parameter** |  | **Observed Assoc.** | |  | **Enhanced Assoc.** | |  | **Interaction Scenarios** |
| --- | --- | --- | --- | --- | --- | --- | --- | --- |
|  |  | **Low Missing** | **High Missing** |  | **Low Missing** | **High Missing** |  |  |
| $\nu_{0}$ |  | 2.14 | 3.08 |  | 1.79 | 2.78 |  | 2.78 |
| $\tau_{0}$ |  | -1.15 | -0.16 |  | 1.15 | 2.19 |  | 2.18 |
| $\tau_{4}$ |  | 3.31 | 2.35 |  | 3.54 | 2.54 |  | 2.55 |
| $\omega_{0}$ |  | -1.95 | -0.84 |  | 0.85 | 2.07 |  | 2.08 |
| $\omega_{4}$ |  | 1.56 | 0.47 |  | 1.69 | 0.44 |  | 0.42 |

**Supplementary Table S3:** Summary of the 26 scenarios considered in the simulation study.

| **Scenario Label** |  | **Cohort sample size** | |  | **Strength of associations** | |  | **Outcome generation interaction** | |  | **Missing data mechanism** | |  | **Percentage unintended missingness** | |  | **Subcohort selection probability** | | |
| --- | --- | --- | --- | --- | --- | --- | --- | --- | --- | --- | --- | --- | --- | --- | --- | --- | --- | --- | --- |
|  |  | **1,000** | **10,000** |  | **Observed** | **Enhanced** |  | **Present** | **Absent** |  | **Independent** | **Dependent** |  | **25%** | **50%** |  | **0.3** | **0.1** | **0.2** |
| *Obsdep1* |  | X |  |  | X |  |  |  | X |  |  | X |  | X |  |  | X |  |  |
| *Obsdep2* |  |  | X |  | X |  |  |  | X |  |  | X |  | X |  |  |  | X |  |
| *Obsdep3* |  |  | X |  | X |  |  |  | X |  |  | X |  | X |  |  |  |  | X |
| *Obsdep4* |  | X |  |  | X |  |  |  | X |  |  | X |  |  | X |  | X |  |  |
| *Obsdep5* |  |  | X |  | X |  |  |  | X |  |  | X |  |  | X |  |  | X |  |
| *Obsdep6* |  |  | X |  | X |  |  |  | X |  |  | X |  |  | X |  |  |  | X |
|  |  |  |  |  |  |  |  |  |  |  |  |  |  |  |  |  |  |  |  |
| *Enhdep1* |  | X |  |  |  | X |  |  | X |  |  | X |  | X |  |  | X |  |  |
| *Enhdep2* |  |  | X |  |  | X |  |  | X |  |  | X |  | X |  |  |  | X |  |
| *Enhdep3* |  |  | X |  |  | X |  |  | X |  |  | X |  | X |  |  |  |  | X |
| *Enhdep4* |  | X |  |  |  | X |  |  | X |  |  | X |  |  | X |  | X |  |  |
| *Enhdep5* |  |  | X |  |  | X |  |  | X |  |  | X |  |  | X |  |  | X |  |
| *Enhdep6* |  |  | X |  |  | X |  |  | X |  |  | X |  |  | X |  |  |  | X |
|  |  |  |  |  |  |  |  |  |  |  |  |  |  |  |  |  |  |  |  |
| *Enhdep5x* |  |  | X |  |  | X |  | X |  |  |  | X |  |  | X |  |  | X |  |
| *Enhdep6x* |  |  | X |  |  | X |  | X |  |  |  | X |  |  | X |  |  |  | X |
|  |  |  |  |  |  |  |  |  |  |  |  |  |  |  |  |  |  |  |  |
| *Obsindep1* | | X |  |  | X |  |  |  | X |  | X |  |  | X |  |  | X |  |  |
| *Obsindep2* | |  | X |  | X |  |  |  | X |  | X |  |  | X |  |  |  | X |  |
| *Obsindep3* | |  | X |  | X |  |  |  | X |  | X |  |  | X |  |  |  |  | X |
| *Obsindep4* | | X |  |  | X |  |  |  | X |  | X |  |  |  | X |  | X |  |  |
| *Obsindep5* | |  | X |  | X |  |  |  | X |  | X |  |  |  | X |  |  | X |  |
| *Obsindep6* | |  | X |  | X |  |  |  | X |  | X |  |  |  | X |  |  |  | X |
|  |  |  |  |  |  |  |  |  |  |  |  |  |  |  |  |  |  |  |  |
| *Enhindep1* | | X |  |  |  | X |  |  | X |  | X |  |  | X |  |  | X |  |  |
| *Enhindep2* | |  | X |  |  | X |  |  | X |  | X |  |  | X |  |  |  | X |  |
| *Enhindep3* | |  | X |  |  | X |  |  | X |  | X |  |  | X |  |  |  |  | X |
| *Enhindep4* | | X |  |  |  | X |  |  | X |  | X |  |  |  | X |  | X |  |  |
| *Enhindep5* | |  | X |  |  | X |  |  | X |  | X |  |  |  | X |  |  | X |  |
| *Enhindep6* | |  | X |  |  | X |  |  | X |  | X |  |  |  | X |  |  |  | X |

**Supplementary Table S4:** Summary statistics for the 26 scenarios, calculated across the 2,000 simulated datasets.

| **Scenario label** |  | **Outcome prevalence** | | **Case-cohort sample size** | |  | **Overall missing** | | | | |
| --- | --- | --- | --- | --- | --- | --- | --- | --- | --- | --- | --- |
|  |  |  |  |  |  |  | **Full cohort** | |  | **Subset** | |
| *Obsdep1* |  | 6.6% | (0.76%) | 347 | (15) |  | 74.2% | (1.41%) |  | 25.4% | (2.34%) |
| *Obsdep2* |  | 6.6% | (0.25%) | 1597 | (37) |  | 88.2% | (0.33%) |  | 25.9% | (1.06%) |
| *Obsdep3* |  | 6.6% | (0.25%) | 2528 | (43) |  | 81.2% | (0.40%) |  | 25.6% | (0.88%) |
| *Obsdep4* |  | 6.7% | (0.76%) | 347 | (15) |  | 82.6% | (1.18%) |  | 50.0% | (2.58%) |
| *Obsdep5* |  | 6.6% | (0.24%) | 1596 | (38) |  | 92.0% | (0.27%) |  | 50.1% | (1.28%) |
| *Obsdep6* |  | 6.6% | (0.24%) | 2528 | (43) |  | 87.4% | (0.33%) |  | 50.1% | (1.00%) |
|  |  |  |  |  |  |  |  |  |  |  |  |
| *Enhdep1* |  | 20.4% | (1.29%) | 443 | (16) |  | 67.5% | (1.47%) |  | 26.5% | (2.06%) |
| *Enhdep2* |  | 20.4% | (0.40%) | 2834 | (45) |  | 79.6% | (0.41%) |  | 28.0% | (0.86%) |
| *Enhdep3* |  | 20.4% | (0.40%) | 3631 | (49) |  | 73.5% | (0.43%) |  | 27.1% | (0.74%) |
| *Enhdep4* |  | 20.4% | (1.27%) | 443 | (16) |  | 78.2% | (1.31%) |  | 50.8% | (2.38%) |
| *Enhdep5* |  | 20.4% | (0.41%) | 2835 | (45) |  | 86.3% | (0.35%) |  | 51.6% | (0.95%) |
| *Enhdep6* |  | 20.4% | (0.41%) | 3632 | (48) |  | 82.2% | (0.38%) |  | 51.1% | (0.82%) |
|  |  |  |  |  |  |  |  |  |  |  |  |
| *Enhdep5x* |  | 20.0% | (0.40%) | 2804 | (44) |  | 86.1% | (0.35%) |  | 50.4% | (0.96%) |
| *Enhdep6x* |  | 20.1% | (0.40%) | 3605 | (47) |  | 82.1% | (0.38%) |  | 50.3% | (0.85%) |
|  |  |  |  |  |  |  |  |  |  |  |  |
| *Obsindep1* |  | 6.7% | (0.79%) | 347 | (15) |  | 74.0% | (1.36%) |  | 25.0% | (2.30%) |
| *Obsindep2* |  | 6.6% | (0.25%) | 1594 | (37) |  | 88.1% | (0.33%) |  | 25.0% | (1.15%) |
| *Obsindep3* |  | 6.6% | (0.26%) | 2530 | (44) |  | 81.0% | (0.39%) |  | 25.0% | (0.84%) |
| *Obsindep4* |  | 6.7% | (0.75%) | 347 | (15) |  | 82.6% | (1.20%) |  | 49.8% | (2.68%) |
| *Obsindep5* |  | 6.6% | (0.24%) | 1595 | (36) |  | 92.0% | (0.28%) |  | 50.0% | (1.30%) |
| *Obsindep6* |  | 6.6% | (0.25%) | 2529 | (43) |  | 87.4% | (0.32%) |  | 50.0% | (0.97%) |
|  |  |  |  |  |  |  |  |  |  |  |  |
| *Enhindep1* |  | 20.3% | (1.25%) | 442 | (15) |  | 66.8% | (1.48%) |  | 25.0% | (2.06%) |
| *Enhindep2* |  | 20.4% | (0.42%) | 2833 | (46) |  | 78.8% | (0.41%) |  | 25.0% | (0.77%) |
| *Enhindep3* |  | 20.4% | (0.40%) | 3629 | (48) |  | 72.8% | (0.44%) |  | 25.0% | (0.70%) |
| *Enhindep4* |  | 20.4% | (1.25%) | 443 | (16) |  | 77.8% | (1.30%) |  | 49.9% | (2.35%) |
| *Enhindep5* |  | 20.4% | (0.41%) | 2834 | (46) |  | 85.8% | (0.35%) |  | 50.0% | (0.93%) |
| *Enhindep6* |  | 20.4% | (0.41%) | 3630 | (48) |  | 81.9% | (0.38%) |  | 50.0% | (0.82%) |

Mean and standard deviation provided for each summary statistics.

# Additional simulation study results

Supplementary material presented in this section provides additional results for the simulation study described in the manuscript.

Figure S1 provides the relative error in estimating the standard error for the target parameter (expressed as a percentage) and S2 provide the model-based standard errors for the target parameter, respectively.


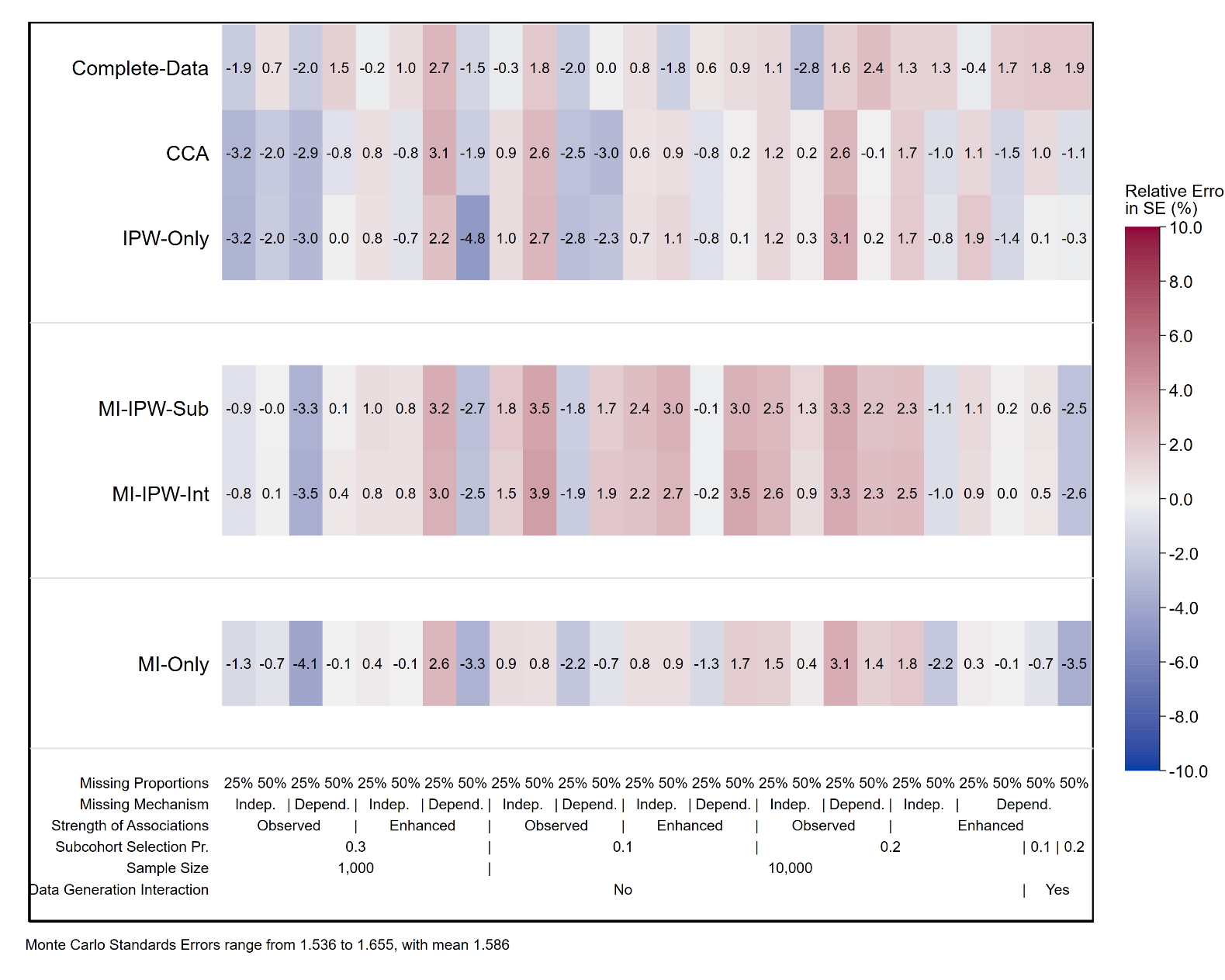


**Figure S1:** Relative error (%) in estimation of the standard error for the target parameter (comparison of empirical and model-based standard error) for each of the 26 simulated scenarios


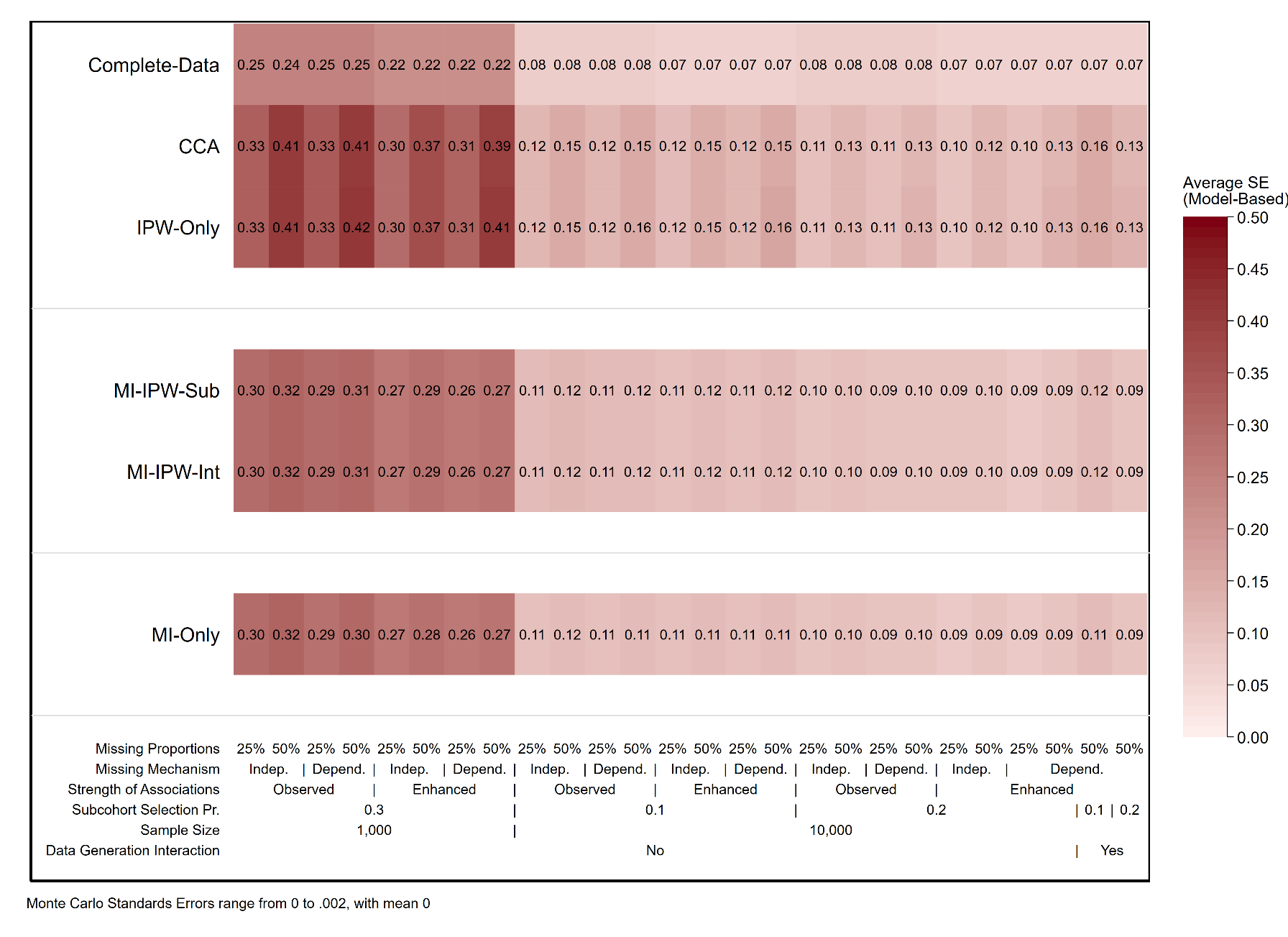


**Figure S2:** Model-based standard error for the analysis approaches across the 2,000 simulated datasets under each scenario
